# Inter- and Intra-Observer Reliability for Colon Capsule CLEansing Assessment and Report

**DOI:** 10.1055/a-2961-4842

**Published:** 2026-09-30

**Authors:** Eimear Gibbons, Syafiq Ismail, Dana AlSharqi, Lisa Keating, Claire Smyth, Richard Farrell, Barry Hall, Orlaith Kelly

**Affiliations:** 1 Department of Gastroenterology 58867 Connolly Hospital BlanchardstownBlanchardstownDublinIreland; 2 Department of Gastroenterology and Clinical MedicineTallaght University HospitalDublinDublinIreland; 3 Department of Gastroenterology 57978 Beaumont HospitalDublinLeinsterIreland

**Keywords:** endoscopy small bowel, capsule endoscopy, quality and logistical aspects, image and data processing, documentation, quality management

## Abstract

A validated scoring system for reporting of colonic cleanliness at colon capsule endoscopy (CCE) is not routinely used to date and great inter-observer variability exists. To ensure appropriate and standardised bowel cleansing reporting, a validated scoring system is of great importance. This would improve the quality of colon capsule reporting and facilitate optimisation of bowel preparation protocols by supporting research reliability. A novel system named Colon Capsule CLEansing Assessment and Report (CC-Clear) has shown superior inter- and intra-observer agreement to the Leighton-Rex scale, an earlier scoring system from 2011. This was a retrospective, single-centre study assessing the intra- and inter-observer reliability of CC-Clear.

**Materials and Methods** Studies were identified retrospectively from the capsule database of a tertiary referral centre. In all, 30 consecutive complete CCE were included. Three expert capsule readers reviewed and reported each examination twice.

**Results and Conclusion** The inter-observer agreement was reported as Kendall’s *W* and was 0.8, *p* < 0.01. An intra-rater reliability analysis was performed between the first and second readings of the three readers. The weighted Cohen’s Kappa showed that there was moderate agreement between the readings of Reader A with κ = 0.52 and *p* < 0.001. For Reader B, the weighted Cohen’s Kappa showed moderate agreement between first and second readings, κ = 0.5 and *p* < 0.001. For Reader C, the weighted Cohen’s Kappa revealed almost perfect agreement between reading with κ = 0.8 and *p* < 0.001. CC-Clear is a simple tool for the CCE reader to utilise and has been shown again here to have excellent inter- and intra-observer reliability.

## Introduction

Colon capsule endoscopy (CCE) has now been established as a viable diagnostic alternative to colonoscopy. 1 It is comparable to colonoscopy in terms of polyp detection. 2 It is safe and has good patient acceptability. 3 4 5 A major drawback, however, is the high rate of re-investigation after CCE. 6 Most often, this is due to failed colonic cleanliness or the need for polypectomy. In terms of colonic cleanliness, a recent meta-analysis of the diagnostic accuracy of CCE versus traditional colonoscopy for polyp detection found adequate colonic cleanliness ranging from 40% to 100% concluding that improvements are required before widespread implementation of CCE into colorectal cancer surveillance programmes. 7 Rosa B et al. in a systematic review and meta-analysis concluded that in addition to the need to improve colon cleansing, there is still no consensus on the preparation protocol to be used. 8

An incomplete colon capsule examination due to failed colonic cleanliness is associated with great inconvenience to both patients and healthcare providers. An incomplete examination often requires further investigation with the same or similar procedure. For the patient, this involves a second and likely extended, bowel preparation to be completed, further time spent in a hospital setting and worry regarding a prolonged investigative pathway. For healthcare systems, repeat procedures exacerbate waiting lists and can increase costs. A validated scoring system for reporting of colonic cleanliness at CCE is not routinely used to date and great inter-observer variability exists. 9 To ensure appropriate and standardised bowel cleansing reporting, a validated scoring system is of great importance. This would improve the quality of colon capsule reporting. It would facilitate optimisation of bowel preparation protocols by supporting research reliability and validity.

A novel system named Colon Capsule CLEansing Assessment and Report (CC-Clear) has shown superior inter- and intra-observer agreement to the Leighton-Rex scale, an earlier scoring system from 2011. 10 11 12 The Leighton-Rex scale is a qualitative scale that divides the colon into five segments: caecum, right colon, transverse, left colon and rectum. Each segment is graded based on the degree of mucosa obscured by faecal matter (‘poor’: mucosa largely obscured, ‘good’: faecal matter does not obscure enough of the mucosa to prevent visualisation of polyps > 5 mm and ‘excellent’: clear image). There is also further scoring that grades the mucosa obscured by bubbles. In CC-Clear, by contrast, the colon is divided into three segments only: right colon, transverse and left colon. Each segment is graded according to the estimated percentage of visualised mucosa. The sum of each segmental score is reported as excellent (8–9), good (6–7) and inadequate if less than or equal to 5. If any one segment scores less than or equal to 5, this results in an inadequate overall classification. To date, studies validating this scoring system have been performed only by the group who created CC-Clear. 10 11

## Methods

### Study Design and Population

This was a retrospective, single-centre study assessing the intra- and inter-observer variability of the novel reporting system, CC-Clear. Studies were identified from the capsule database of a tertiary referral centre. The first 30 complete colon capsule examinations were included in the final analysis. All colon capsules included in the final analysis had the standard split-dose bowel preparation used in the department ( Table 1 ). All patients were pre-assessed by a clinical nurse specialist prior to ingestion of colon capsule. Three expert capsule readers reviewed each selected examination twice.

**Table 1:** Bowel preparation regimen.

1 week before	Stop iron tablets
2 days before	4 × 7.5 mg Senna tablets at 7 pm
1 day before	Clear liquid diet throughout the day (includes black tea, black coffee, clear broth, soft drinks, jelly-nothing, blue or red)
	7 pm first dose of Moviprep A+B in 1 L water. This should be followed by at least another 500 mL of water or another clear fluid
	Clear fluids only until midnight
Test day (morning)	Essential medications may be taken with sips of water
	6 am second dose Moviprep A+B in 1 L water. This should be followed by another 500 mL water
	On arrival at the department, the recorder is attached and the capsule is swallowed. Participants wait in the department for up to 30 min to allow capsule to pass into the small bowel. The first booster is then given
	The first booster is prepared by member of team: 750 mL of Moviprep plus 750 mL of water along with 50 mL Gastrograffin
	From November 2022, cases also received the additional 15 mL castor oil at this point
	The remaining 250 mL Moviprep are kept for second booster in the empty water bottle
	Participants can now drink clear fluids freely
Test day (3 h after first booster)	Second booster to be taken: 250 mL Moviprep plus 250 mL water
	Participant can now drink and eat as normal and take regular medication
Test day (2 h after second booster)	If capsule has not passed, rectal suppository (Dulcolax) to be taken
After the test	Remove the belt when the capsule passes or the battery runs out
	Return the recorder and sensor belt to department in envelope provided

### Procedure Details

All procedures were completed using the PillCam COLON2 system (Medtronic, Minneapolis, MN, USA). All patients received four 12 mg Senakot tablets to take two days prior to capsule ( Table 1 ). They completed their preparation prior to attending the department with two-litre, split-dose Moviprep. Patients were advised to take the first litre the evening before capsule, typically around 7 pm, with the second litre taken the morning of the procedure at 6 am. If the capsule remained in the stomach at 30 min post ingestion, all patients were given oral metoclopromide. The booster regime included 50 mL gastrograffin with a further 750 mL Moviprep and was taken when the capsule reached the small bowel. A further 250 mL Moviprep was given 3 hours later if the capsule had not passed. The patients were provided with a Dulcolax suppository to take after 8 hours if the capsule was still in transit. In this department, prokinetics are given only if a patient has known risk factors for slow gastric transit. If risk of delayed emptying is identified, patients receive 10 mg Metoclopromide orally.

### Data Analysis

Patient demographics including age, gender and indication for CCE were captured. Colonic transit was defined as the time from first caecal image until capsule excretion. Total examination time was the interval between capsule ingestion and excretion, or battery life in those with incomplete examinations. Differences were considered significant with *p* -values <0.05. Inter-observer agreement was reported as Kendall’s *W* , and intra-observer agreement was calculated as Cohen’s Kappa.

## Results

### Patient Demographics

In all, 30 complete colon capsule examinations were included in the final analysis ( Table 2 ). The median age was 57 (range 34–84). Overall, 70% of patients were women. Indications for CCE included failed colonoscopy, constipation, abdominal pain, anaemia and surveillance.

**Table 2:** Clinical characteristics of patients.

Characteristic	Total
No.	30
Age, years	
Median	57
Range	34–84
Gender	
Male	9
Female	21
Indications for CCE	
Failed colonoscopy	20/30
Constipation	6/30
Abdominal pain	2/30
Anaemia	1/30
Surveillance	1/30
Mean total transit (min)	296

### Inter-observer Reliability

The overall CC-Clear cleansing classifications were as follows ( Table 3 ). Reader A considered the quality of bowel preparation excellent in 56.6% ( *n* = 17), good in 20% ( *n* = 6) and inadequate in 23% ( *n* = 7). Reader B considered 40% ( *n* = 12) excellent, 46.6% ( *n* = 14) good and 13.4% ( *n* = 4) inadequate. Reader C considered 43.5% ( *n* = 13) excellent, 36.5% ( *n* = 11) good and 20% ( *n* = 6) inadequate. The inter-observer agreement was reported as Kendall’s *W* and was as follows: overall classification Kendall’s *W* 0.8, *p* < 0.01; right colon Kendall’s *W* 0.76, *p* < 0.01; transverse colon Kendall’s *W* 0.78, *p* < 0.01; and left colon Kendall’s *W* 0.58, *p* = 0.08. When a two point scale was applied (adequate or inadequate), Kendall’s *W* was 0.70, *p* < 0.01.

**Table 3:** Colon Capsule Cleansing Assessment and Report scoring per reader and interobserver agreement.

No.	Overall Reader 1	Overall Reader 2	Overall Reader 3
1	Excellent	Good	Excellent
2	Excellent	Good	Excellent
3	Excellent	Excellent	Excellent
4	Inadequate	Excellent	Excellent
5	Excellent	Excellent	Excellent
6	Excellent	Excellent	Excellent
7	Good	Good	Excellent
8	Excellent	Excellent	Excellent
9	Good	Good	Good
10	Inadequate	Inadequate	Inadequate
11	Excellent	Excellent	Excellent
12	Good	Good	Good
13	Good	Good	Good
14	Excellent	Excellent	Excellent
15	Excellent	Excellent	Excellent
16	Inadequate	Inadequate	Inadequate
17	Excellent	Good	Good
18	Excellent	Excellent	Good
19	Inadequate	Good	Good
20	Good	Inadequate	Inadequate
21	Excellent	Good	Inadequate
22	Good	Good	Good
23	Inadequate	Good	Inadequate
24	Excellent	Good	Inadequate
25	Inadequate	Good	Excellent
26	Excellent	Good	Excellent
27	Excellent	Excellent	Excellent
28	Excellent	Excellent	Excellent
29	Excellent	Excellent	Excellent
30	Inadequate	Inadequate	Inadequate

### Intra-observer Reliability

On first reading, reader A considered the quality of bowel preparation excellent in 56.6% ( *n* = 17), good in 20% ( *n* = 6) and inadequate in 23% ( *n* = 7). On second reading, reader A considered 50% ( *n* = 15) excellent, 26.6% ( *n* = 8) good, and 23.4% ( *n* = 7) inadequate. On first reading, Reader B considered 40% ( *n* = 12) excellent, 46.6% ( *n* = 14) good and 13.4% ( *n* = 4) inadequate. On second reading, reader B considered 43.4% ( *n* = 13) excellent, 43.4% ( *n* = 13) good and 13.4% ( *n* = 4) inadequate. On first reading, Reader C considered 43.5% ( *n* = 13) excellent, 36.5% ( *n* = 11) good and 20% ( *n* = 6) inadequate. On second reading, reader C considered 50% ( *n* = 15) excellent, 26.6% ( *n* = 8) good and 23.3% ( *n* = 7) inadequate. Table 4 summarises the scoring each reader applied to the procedures numbered 1–30.

**Table 4:** Colon Capsule Cleansing Assessment and Report scoring per reader.

No.	Reader A-1st reading	Reader A-2nd reading	Reader B-1st reading	Reader B-2nd reading	Reader C-1st reading	Reader C-2nd reading
1	Excellent	Excellent	Good	Excellent	Excellent	Excellent
2	Excellent	Good	Good	Good	Excellent	Good
3	Excellent	Excellent	Excellent	Excellent	Excellent	Excellent
4	Inadequate	Inadequate	Excellent	Good	Excellent	Excellent
5	Excellent	Excellent	Excellent	Excellent	Excellent	Excellent
6	Excellent	Excellent	Excellent	Excellent	Excellent	Excellent
7	Good	Good	Good	Good	Excellent	Excellent
8	Excellent	Excellent	Excellent	Excellent	Excellent	Excellent
9	Good	Inadequate	Good	Good	Good	Good
10	Inadequate	Inadequate	Inadequate	Inadequate	Inadequate	Inadequate
11	Excellent	Excellent	Excellent	Excellent	Excellent	Excellent
12	Good	Excellent	Good	Good	Good	Good
13	Good	Good	Good	Good	Good	Good
14	Excellent	Excellent	Excellent	Excellent	Excellent	Excellent
15	Excellent	Excellent	Excellent	Excellent	Excellent	Excellent
16	Inadequate	Inadequate	Inadequate	Inadequate	Inadequate	Inadequate
17	Excellent	Excellent	Good	Excellent	Good	Good
18	Excellent	Excellent	Excellent	Excellent	Good	Good
19	Inadequate	Inadequate	Good	Good	Good	Good
20	Good	Good	Inadequate	Good	Inadequate	Inadequate
21	Excellent	Good	Good	Inadequate	Inadequate	Inadequate
22	Good	Good	Good	Good	Good	Good
23	Inadequate	Inadequate	Good	Good	Inadequate	Inadequate
24	Excellent	Good	Good	Good	Inadequate	Inadequate
25	Inadequate	Good	Good	Good	Excellent	Excellent
26	Excellent	Excellent	Good	Excellent	Excellent	Excellent
27	Excellent	Excellent	Excellent	Good	Excellent	Excellent
28	Excellent	Excellent	Excellent	Excellent	Excellent	Excellent
29	Excellent	Excellent	Excellent	Excellent	Excellent	Excellent
30	Inadequate	Inadequate	Inadequate	Inadequate	Inadequate	Inadequate

An intra-rater reliability analysis was performed between the first and second readings of Reader A. The weighted Cohen’s Kappa showed that there was moderate agreement between the readings with κ = 0.52 and a *p* -value of <0.001. For Reader B, the weighted Cohen’s Kappa showed that there was a moderate agreement between first and second readings with κ = 0.5 and a *p* -value of <0.001. For Reader C, the weighted Cohen’s Kappa showed that there was an almost perfect agreement between reading with κ = 0.8 and a *p* -value of <0.001.

## Discussion

It is felt that the commonly used Boston Bowel Preparation (BBP) scale for colonoscopy is not appropriate for direct application to CCE reporting, for two main reasons. 10 13 Firstly, this scale is applied after washing and suctioning at colonoscopy, two tools not available at CCE. And secondly, the scale is devised for the use of conventional colonoscopy images, and not images captured at CCE.

The most commonly used scoring system for CCE, to date, has been the Leighton Rex score developed in 2011. 12 This classification relies on the readers’ subjective impression for qualitative assessment and is cumbersome for the reader. Anatomic land marking can be a challenge for the reader at colon capsule endoscopy further limiting the applicability and rigor of this scale which sees the colon divided into five sections. It did not achieve high inter-observer agreement in early studies. 12

CC-Clear is a user-friendly and practical tool for CCE readers. The first study to report on its inter- and intra-observer reliability, as well as provide comparison to the Leighton Rex scale, was very encouraging, with results similar to this study. 10 It included 58 consecutive colon capsule examinations, read by two expert readers. The inter-observer agreement of CC-Clear was excellent (Kendall’s *W* , 0.911; *p* < 0.01) and higher than the Leighton-Rex scale (Kendall’s *W* , 0.44). It also reported excellent intra-observer agreement (Kendall’s *W* , 0.939; *p* < 0.01 for reader A, Kendall’s *W* , 0.947, *p* < 0.01 for reader B).

Agreement on colonic cleansing quality has been shown to be poor among clinicians. 9 13 One study showed that readers with more experience had improved polyp detection rates compared to readers who were new to reporting colon capsules, but noted that this improvement was not seen with colonic cleanliness reporting. 9 They concluded that colonic cleanliness reporting needed to be improved to ultimately increase the reliability of CCE. 9 It is established that at colonoscopy, clear guidelines and training in reporting of colonic cleanliness can improve inter-observer reliability. 13

We acknowledge that our study has some limitations. This was a single centre, retrospective study and there were only a small number of capsule examinations included in the final analysis.

There is currently widespread variation in preparation protocols used in clinical practise and a need for large, prospective studies comparing these protocols. 8 14 CC-Clear is a simple tool for the CCE reader to utilise and has been validated again here to have excellent inter- and intra-observer reliability.

## References

[1] Spada C Hassan C Bellini D Imaging alternatives to colonoscopy: CT colonography and colon capsule. European Society of Gastrointestinal Endoscopy (ESGE) and European Society of Gastrointestinal and Abdominal Radiology (ESGAR) guideline - update 2020 Eur Radiol 2021 31 2967 2982 10.1007/s00330-020-07413-4 33104846

[2] Spada C Pasha S F Gross S A Accuracy of first- and second-generation colon capsules in endoscopic detection of colorectal polyps: a systematic review and meta-analysis Clin Gastroenterol Hepatol 2016 14 INF INF 10.1016/j.cgh.2016.04.038 27165469

[3] Rezapour M Amadi C Gerson L B Retention associated with video capsule endoscopy: systematic review and meta-analysis Gastrointest Endosc 2017 85 INF INF 10.1016/j.gie.2016.12.024 28069475

[4] Yung D E Plevris J N Koulaouzidis A Short article: aspiration of capsule endoscopes: a comprehensive review of the existing literature Eur J Gastroenterol Hepatol 2017 29 428 434 10.1097/meg.0000000000000821 28253209

[5] Deding U Cortegoso Valdivia P Koulaouzidis A Patient-reported outcomes and preferences for colon capsule endoscopy and colonoscopy: a systematic review with meta-analysis Diagnostics 2021 11 10.3390/diagnostics11091730 PMC8468090 34574071

[6] MacLeod C Hudson J Brogan M ScotCap – a large observational cohort study Color Dis 2022 24 411 421 10.1111/codi.16029 PMC9305214 34935278

[7] Kjølhede T Ølholm A M Kaalby L Diagnostic accuracy of capsule endoscopy compared with colonoscopy for polyp detection: systematic review and meta-analyses Endoscopy 2021 53 713 721 10.1055/a-1249-3938 32858753

[8] Rosa B Donato H Curdia Goncalves T What is the optimal bowel preparation for capsule colonoscopy and pan-intestinal capsule endoscopy? A systematic review and meta-analysis Dig Dis Sci 2023 68 4418 4431 10.1007/s10620-023-08133-7 PMC10635919 37833441

[9] Buijs M M Kroijer R Kobaek-Larsen M Intra and inter-observer agreement on polyp detection in colon capsule endoscopy evaluations United Eur Gastroenterol J 2018 6 1563 1568 10.1177/2050640618798182 PMC6297930 30574327

[10] de Sousa M R Arieira C Boal Carvalho P Colon capsule CLEansing assessment and report (CC-CLEAR): a new approach for evaluation of the quality of bowel preparation in capsule colonoscopy Gastrointest Endosc 2021 93 212 223 10.1016/j.gie.2020.05.062 32534054

[11] de Sousa M R Chálim Rebelo C Sousa-Pinto B CC-CLEAR (colon capsule cleansing assessment and report): the novel scale to evaluate the clinical impact of bowel preparation in capsule colonoscopy - a multicentric validation study Scand J Gastroenterol 2022 57 625 632 10.1080/00365521.2022.2026463 35068293

[12] Leighton J A Rex D K A grading scale to evaluate colon cleansing for the PillCam COLON capsule: a reliability study Endoscopy 2011 43 123 127 10.1055/s-0030-1255916 21038293

[13] Lai E J Calderwood A H Doros G The Boston bowel preparation scale: a valid and reliable instrument for colonoscopy-oriented research Gastrointest Endosc 2009 69 620 625 10.1016/j.gie.2008.05.057 PMC2763922 19136102

[14] Bjoersum-Meyer T Skonieczna-Zydecka K Cortegoso Valdivia P Efficacy of bowel preparation regimens for colon capsule endoscopy: a systematic review and meta-analysis Endosc Int Open 2021 9 E1658 E1673 10.1055/a-1529-5814 PMC8589531 34790528

